# Ethyl 8-(2,4-di­chloro­phen­yl)-6-methyl-1,2,4-triazolo[1,5-*a*]pyridine-7-carboxyl­ate

**DOI:** 10.1107/S160053681303033X

**Published:** 2013-11-20

**Authors:** Yang Li, Chen Sun, Ran Zhang

**Affiliations:** a School of Chemical Engineering, Taishan Medical University, Taian 271016, People’s Republic of China

## Abstract

In the title compound, C_16_H_13_Cl_2_N_3_O_2_, the carboxyl­ate group and the benzene ring attached to the central 1,2,4-triazolo[1,5-*a*]pyridine bicycle are twisted from its mean plane by 55.6 (1) and 72.6 (1)°, respectively. In the crystal, weak C—H⋯O inter­actions link the mol­ecules into zigzag chains propagating in [100].

## Related literature

For applications of [1,2,4]triazolo[1,5-*a*]pyridine derivatives, see: Luo & Hu (2006 ▶); Liu & Hu (2002 ▶). For details of the synthesis, see: Jones & Sliskovic (1983 ▶); Wang *et al.* (2003 ▶); Ge *et al.* (2009 ▶); Jia *et al.* (2010 ▶). For standard bond lengths, see: Allen *et al.* (1987 ▶).
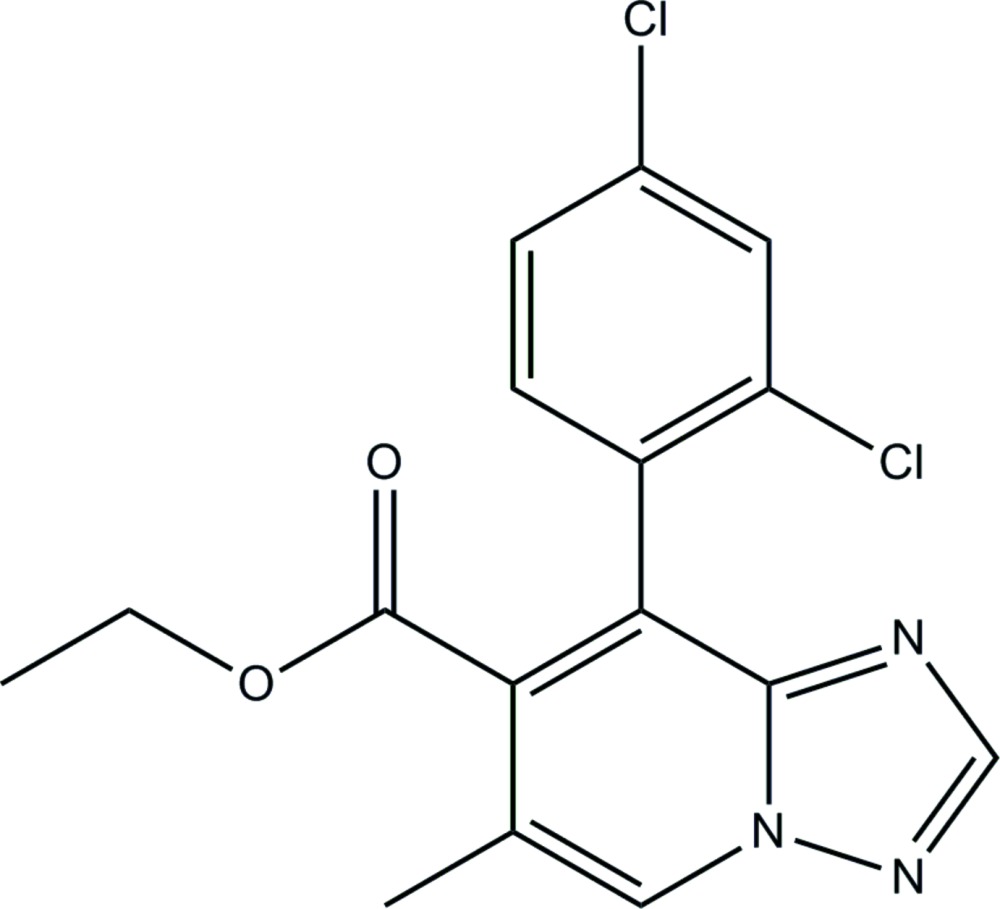


## Experimental

### 

#### Crystal data

C_16_H_13_Cl_2_N_3_O_2_
*M* *_r_* = 350.19
Orthorhombic, 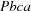

*a* = 14.693 (2) Å
*b* = 13.531 (2) Å
*c* = 16.347 (2) Å
*V* = 3250.0 (8) Å^3^
*Z* = 8
Mo *K*α radiation
μ = 0.41 mm^−1^
*T* = 298 K
0.33 × 0.26 × 0.21 mm

#### Data collection

Brucker SMART APEXII CCD area-detector diffractometer
Absorption correction: multi-scan (*SADABS*; Bruker, 1999 ▶) *T*_min_ = 0.876, *T*_max_ = 0.919
15766 measured reflections
2860 independent reflections
2206 reflections with *I* > 2σ(*I*)
*R*_int_ = 0.090

#### Refinement

*R*[*F* ^2^ > 2σ(*F* ^2^)] = 0.055
*wR*(*F* ^2^) = 0.170
*S* = 1.07
2860 reflections
210 parameters
H-atom parameters constrained
Δρ_max_ = 0.42 e Å^−3^
Δρ_min_ = −0.33 e Å^−3^

### 

Data collection: *SMART* (Bruker, 1998 ▶); cell refinement: *SAINT* (Bruker, 1999 ▶); data reduction: *SAINT*; program(s) used to solve structure: *SHELXS97* (Sheldrick, 2008 ▶); program(s) used to refine structure: *SHELXL97* (Sheldrick, 2008 ▶); molecular graphics: *SHELXTL* (Sheldrick, 2008 ▶); software used to prepare material for publication: *SHELXTL*.

## Supplementary Material

Crystal structure: contains datablock(s) 120712a, I. DOI: 10.1107/S160053681303033X/cv5435sup1.cif

Structure factors: contains datablock(s) I. DOI: 10.1107/S160053681303033X/cv5435Isup2.hkl

Supplementary material file. DOI: 10.1107/S160053681303033X/cv5435Isup3.cml

Additional supplementary materials:  crystallographic information; 3D view; checkCIF report

## Figures and Tables

**Table 1:** Hydrogen-bond geometry (Å, °)

*D*—H⋯*A*	*D*—H	H⋯*A*	*D*⋯*A*	*D*—H⋯*A*
C7—H7⋯O1^i^	0.93	2.55	3.296 (4)	137

Symmetry code: (i) 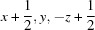.

**Fig. 1.**
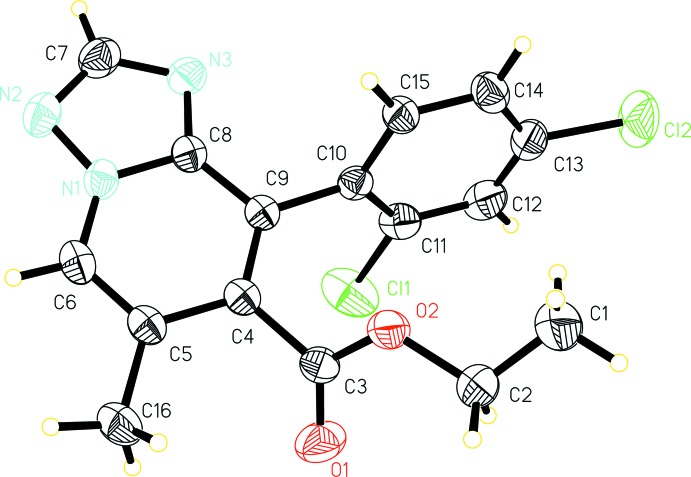
View of (I) with displacement ellipsoids drawn at the 30% probability level.

## supplementary crystallographic information

### 1. Comment

The [1,2,4]triazolo[1,5-*a*]pyridine derivatives exhibit antifungal, anticancer and anti-inflammatory activities (Liu & Hu, 2002; Luo & Hu, 2006). However, only small number of [1,2,4]triazolo[1,5-*a*]pyridines is known. The commonly used synthetic methods are the annulation of 1,2,4-triazole ring starting with amino substituted pyridines by a multistep procedure (Jones & Sliskovic, 1983). Recently, imidazo[1,5-*a*]pyridines, pyrazolo[1,5-*a*]pyridines, imidazo[1,2-*a*]pyridines and indolizines have been synthesized in our group wityh the use of a novel tandem reaction (Wang *et al.*, 2003; Ge *et al.*, 2009; Jia *et al.*, 2010). We tried to extend this reaction to synthesize the [1,2,4]triazolo[1,5-*a*]pyridine heterocycles and obtained the title compound (I). Herewith we present its crystal structure.

In (I) (Fig. 1), all bond lengths and angles are normal (Allen *et al.*, 1987). The carboxylate group and benzene ring attached to the central [1,2,4]triazolo[1,5-*a*]pyridine bicycle are twisted from its mean plane at 55.6 (1) and 72.6 (1)°, respectively. In the crystal, weak intermolecular C—H···O interactions (Table 1) link molecules into zigzag chains propagated in [100].

### 2. Experimental

(2,4-Dichlorophenyl)(1*H*-1,2,4-triazol-5-yl)methanone (6 mmol), ethyl 4-bromo-3-methylbut-2-enoate (12 mmol), potassium carbonate (1.8 g, 13.2 mmol) and DMF (30 ml) were added to a 100 ml round-bottomed flask. The reaction system was stirred for 8 h. Then the mixture was poured into water (200 ml) and extracted with dichloromethane (3 *x* 50 ml). Organic layers were combined and dried over anhydrous Na_2_SO_4_, then filtered. By rotary evaporation, the mixture was concentrated. After that, these crude products were depurated by using column chromatography in 76% isolated yield. Crystals suitable for X-ray diffraction analysis were obtained by slow evaporation of a solution of the title compound in a hexane/ethyl acetate mixture (3:1 *v*/*v*) at room temperature over a period of one week.

### 3. Refinement

All H atoms were found on difference maps, but placed in idealized positions (C—H = 0.93–0.97 Å), and included in the final cycles of refinement using a riding model, with *U*_iso_(H) = 1.2*U*_eq_(C) and 1.5*U*_eq_(C) for the methyl H atoms.

### Crystal data

**Table:**

C_16_H_13_Cl_2_N_3_O_2_	*F*(000) = 1440
*M**_r_* = 350.19	*D*_x_ = 1.431 Mg m^−^^3^
Orthorhombic, *P**b**c**a*	Mo *K*α radiation, λ = 0.71073 Å
Hall symbol: -P 2ac 2ab	Cell parameters from 5439 reflections
*a* = 14.693 (2) Å	θ = 2.4–26.6°
*b* = 13.531 (2) Å	µ = 0.41 mm^−^^1^
*c* = 16.347 (2) Å	*T* = 298 K
*V* = 3250.0 (8) Å^3^	Block, colourless
*Z* = 8	0.33 × 0.26 × 0.21 mm

### Data collection

**Table:**

Brucker SMART APEXII CCD area-detector diffractometer	2860 independent reflections
Radiation source: fine-focus sealed tube	2206 reflections with *I* > 2σ(*I*)
Graphite monochromator	*R*_int_ = 0.090
phi and ω scans	θ_max_ = 25.0°, θ_min_ = 2.4°
Absorption correction: multi-scan (*SADABS*; Bruker, 1999)	*h* = −15→17
*T*_min_ = 0.876, *T*_max_ = 0.919	*k* = −14→16
15766 measured reflections	*l* = −19→19

### Refinement

**Table:**

Refinement on *F*^2^	Primary atom site location: structure-invariant direct methods
Least-squares matrix: full	Secondary atom site location: difference Fourier map
*R*[*F*^2^ > 2σ(*F*^2^)] = 0.055	Hydrogen site location: inferred from neighbouring sites
*wR*(*F*^2^) = 0.170	H-atom parameters constrained
*S* = 1.07	*w* = 1/[σ^2^(*F*_o_^2^) + (0.081*P*)^2^ + 1.792*P*] where *P* = (*F*_o_^2^ + 2*F*_c_^2^)/3
2860 reflections	(Δ/σ)_max_ < 0.001
210 parameters	Δρ_max_ = 0.42 e Å^−^^3^
0 restraints	Δρ_min_ = −0.33 e Å^−^^3^

### Special details

**Table:**

Geometry. All e.s.d.'s (except the e.s.d. in the dihedral angle between two l.s. planes) are estimated using the full covariance matrix. The cell e.s.d.'s are taken into account individually in the estimation of e.s.d.'s in distances, angles and torsion angles; correlations between e.s.d.'s in cell parameters are only used when they are defined by crystal symmetry. An approximate (isotropic) treatment of cell e.s.d.'s is used for estimating e.s.d.'s involving l.s. planes.
Refinement. Refinement of *F*^2^ against ALL reflections. The weighted *R*-factor *wR* and goodness of fit *S* are based on *F*^2^, conventional *R*-factors *R* are based on *F*, with *F* set to zero for negative *F*^2^. The threshold expression of *F*^2^ > σ(*F*^2^) is used only for calculating *R*-factors(gt) *etc*. and is not relevant to the choice of reflections for refinement. *R*-factors based on *F*^2^ are statistically about twice as large as those based on *F*, and *R*- factors based on ALL data will be even larger.

### Fractional atomic coordinates and isotropic or equivalent isotropic displacement parameters (Å^2^)

**Table:**

	*x*	*y*	*z*	*U*_iso_*/*U*_eq_	
Cl1	0.05484 (8)	0.40274 (8)	0.16648 (8)	0.1076 (5)	
Cl2	0.19892 (9)	0.61061 (7)	−0.08078 (8)	0.1131 (5)	
N1	0.26136 (16)	0.13047 (17)	0.22123 (12)	0.0520 (6)	
N2	0.32941 (18)	0.1240 (2)	0.27788 (15)	0.0667 (7)	
N3	0.33128 (17)	0.27522 (19)	0.21631 (14)	0.0597 (6)	
O1	−0.01914 (15)	0.1842 (2)	0.05658 (15)	0.0854 (8)	
O2	0.09633 (14)	0.21635 (17)	−0.02602 (11)	0.0703 (6)	
C1	0.0817 (4)	0.3050 (5)	−0.1491 (3)	0.138 (2)	
H1A	0.1140	0.3576	−0.1224	0.207*	
H1B	0.0391	0.3323	−0.1874	0.207*	
H1C	0.1242	0.2634	−0.1775	0.207*	
C2	0.0347 (3)	0.2488 (4)	−0.0903 (2)	0.0953 (13)	
H2A	0.0074	0.1916	−0.1164	0.114*	
H2B	−0.0137	0.2883	−0.0667	0.114*	
C3	0.06111 (18)	0.19142 (19)	0.04424 (16)	0.0498 (6)	
C4	0.13260 (17)	0.17205 (19)	0.10725 (15)	0.0451 (6)	
C5	0.13144 (18)	0.07882 (19)	0.14927 (16)	0.0494 (6)	
C6	0.19780 (19)	0.0596 (2)	0.20462 (17)	0.0558 (7)	
H6	0.2000	−0.0012	0.2310	0.067*	
C7	0.3669 (2)	0.2128 (3)	0.27135 (19)	0.0687 (9)	
H7	0.4161	0.2311	0.3038	0.082*	
C8	0.26346 (18)	0.22155 (19)	0.18455 (15)	0.0478 (6)	
C9	0.19725 (16)	0.24312 (18)	0.12376 (15)	0.0439 (6)	
C10	0.20186 (17)	0.33997 (18)	0.08023 (15)	0.0460 (6)	
C11	0.1376 (2)	0.4141 (2)	0.09152 (19)	0.0597 (7)	
C12	0.1379 (2)	0.4988 (2)	0.0427 (2)	0.0717 (9)	
H12	0.0942	0.5477	0.0500	0.086*	
C13	0.2031 (2)	0.5087 (2)	−0.0156 (2)	0.0652 (8)	
C14	0.2709 (2)	0.4403 (2)	−0.02570 (17)	0.0608 (7)	
H14	0.3167	0.4501	−0.0641	0.073*	
C15	0.26961 (18)	0.3567 (2)	0.02229 (16)	0.0521 (7)	
H15	0.3154	0.3099	0.0158	0.062*	
C16	0.0598 (2)	0.0018 (2)	0.1339 (2)	0.0667 (8)	
H16A	0.0770	−0.0588	0.1604	0.100*	
H16B	0.0542	−0.0093	0.0761	0.100*	
H16C	0.0026	0.0241	0.1554	0.100*	

### Atomic displacement parameters (Å^2^)

**Table:**

	*U*^11^	*U*^22^	*U*^33^	*U*^12^	*U*^13^	*U*^23^
Cl1	0.0972 (8)	0.0803 (7)	0.1453 (10)	0.0092 (5)	0.0583 (7)	−0.0185 (6)
Cl2	0.1464 (10)	0.0607 (6)	0.1323 (10)	−0.0150 (6)	−0.0448 (8)	0.0396 (6)
N1	0.0569 (13)	0.0554 (13)	0.0438 (11)	0.0063 (11)	0.0013 (10)	0.0036 (10)
N2	0.0705 (16)	0.0752 (18)	0.0544 (14)	0.0091 (14)	−0.0085 (12)	0.0073 (12)
N3	0.0583 (14)	0.0654 (15)	0.0555 (13)	−0.0044 (11)	−0.0075 (11)	−0.0013 (11)
O1	0.0490 (13)	0.130 (2)	0.0777 (15)	−0.0008 (13)	0.0030 (11)	0.0062 (15)
O2	0.0600 (12)	0.1014 (17)	0.0494 (11)	−0.0223 (11)	−0.0043 (9)	0.0141 (10)
C1	0.120 (4)	0.192 (6)	0.102 (3)	−0.071 (4)	−0.037 (3)	0.068 (4)
C2	0.091 (3)	0.118 (3)	0.077 (2)	−0.042 (2)	−0.033 (2)	0.035 (2)
C3	0.0508 (16)	0.0464 (14)	0.0523 (14)	−0.0042 (11)	0.0019 (12)	−0.0053 (11)
C4	0.0476 (14)	0.0442 (14)	0.0436 (13)	0.0007 (11)	0.0073 (11)	−0.0030 (10)
C5	0.0534 (15)	0.0442 (14)	0.0507 (14)	−0.0001 (11)	0.0123 (12)	−0.0022 (11)
C6	0.0678 (18)	0.0448 (14)	0.0549 (15)	0.0036 (13)	0.0116 (14)	0.0056 (12)
C7	0.0627 (19)	0.086 (2)	0.0576 (18)	0.0003 (16)	−0.0104 (15)	−0.0001 (16)
C8	0.0521 (15)	0.0480 (15)	0.0435 (13)	0.0012 (11)	0.0037 (11)	−0.0013 (11)
C9	0.0460 (14)	0.0429 (13)	0.0428 (13)	0.0010 (11)	0.0041 (10)	−0.0021 (10)
C10	0.0474 (14)	0.0434 (14)	0.0473 (14)	−0.0026 (11)	−0.0062 (11)	−0.0045 (11)
C11	0.0558 (16)	0.0466 (16)	0.0765 (19)	0.0008 (12)	0.0009 (14)	−0.0113 (13)
C12	0.067 (2)	0.0407 (16)	0.107 (3)	0.0075 (13)	−0.0178 (19)	−0.0072 (16)
C13	0.077 (2)	0.0452 (16)	0.074 (2)	−0.0100 (15)	−0.0236 (17)	0.0054 (14)
C14	0.0716 (19)	0.0550 (17)	0.0559 (16)	−0.0131 (14)	−0.0018 (14)	0.0030 (13)
C15	0.0542 (16)	0.0476 (15)	0.0545 (15)	−0.0030 (12)	−0.0005 (12)	0.0026 (12)
C16	0.0708 (19)	0.0487 (16)	0.081 (2)	−0.0097 (14)	0.0069 (16)	−0.0006 (15)

### Geometric parameters (Å, º)

**Table:**

Cl1—C11	1.733 (3)	C4—C5	1.437 (4)
Cl2—C13	1.743 (3)	C5—C6	1.355 (4)
N1—C6	1.365 (4)	C5—C16	1.502 (4)
N1—N2	1.366 (3)	C6—H6	0.9300
N1—C8	1.371 (3)	C7—H7	0.9300
N2—C7	1.326 (4)	C8—C9	1.421 (4)
N3—C8	1.338 (3)	C9—C10	1.493 (4)
N3—C7	1.341 (4)	C10—C11	1.389 (4)
O1—C3	1.200 (3)	C10—C15	1.393 (4)
O2—C3	1.304 (3)	C11—C12	1.398 (4)
O2—C2	1.455 (4)	C12—C13	1.357 (5)
C1—C2	1.406 (5)	C12—H12	0.9300
C1—H1A	0.9600	C13—C14	1.371 (4)
C1—H1B	0.9600	C14—C15	1.376 (4)
C1—H1C	0.9600	C14—H14	0.9300
C2—H2A	0.9700	C15—H15	0.9300
C2—H2B	0.9700	C16—H16A	0.9600
C3—C4	1.494 (4)	C16—H16B	0.9600
C4—C9	1.378 (4)	C16—H16C	0.9600
			
C6—N1—N2	126.2 (2)	N3—C7—H7	121.2
C6—N1—C8	124.0 (2)	N3—C8—N1	109.6 (2)
N2—N1—C8	109.7 (2)	N3—C8—C9	132.1 (2)
C7—N2—N1	101.1 (2)	N1—C8—C9	118.4 (2)
C8—N3—C7	102.1 (3)	C4—C9—C8	117.7 (2)
C3—O2—C2	117.9 (2)	C4—C9—C10	123.4 (2)
C2—C1—H1A	109.5	C8—C9—C10	118.9 (2)
C2—C1—H1B	109.5	C11—C10—C15	117.3 (3)
H1A—C1—H1B	109.5	C11—C10—C9	122.6 (2)
C2—C1—H1C	109.5	C15—C10—C9	120.0 (2)
H1A—C1—H1C	109.5	C10—C11—C12	120.9 (3)
H1B—C1—H1C	109.5	C10—C11—Cl1	120.5 (2)
C1—C2—O2	110.6 (3)	C12—C11—Cl1	118.6 (2)
C1—C2—H2A	109.5	C13—C12—C11	119.0 (3)
O2—C2—H2A	109.5	C13—C12—H12	120.5
C1—C2—H2B	109.5	C11—C12—H12	120.5
O2—C2—H2B	109.5	C12—C13—C14	122.0 (3)
H2A—C2—H2B	108.1	C12—C13—Cl2	118.9 (3)
O1—C3—O2	124.0 (3)	C14—C13—Cl2	119.1 (3)
O1—C3—C4	124.1 (3)	C13—C14—C15	118.5 (3)
O2—C3—C4	111.9 (2)	C13—C14—H14	120.8
C9—C4—C5	121.8 (2)	C15—C14—H14	120.8
C9—C4—C3	119.8 (2)	C14—C15—C10	122.1 (3)
C5—C4—C3	118.4 (2)	C14—C15—H15	119.0
C6—C5—C4	118.6 (2)	C10—C15—H15	119.0
C6—C5—C16	118.8 (3)	C5—C16—H16A	109.5
C4—C5—C16	122.5 (3)	C5—C16—H16B	109.5
C5—C6—N1	119.4 (2)	H16A—C16—H16B	109.5
C5—C6—H6	120.3	C5—C16—H16C	109.5
N1—C6—H6	120.3	H16A—C16—H16C	109.5
N2—C7—N3	117.6 (3)	H16B—C16—H16C	109.5
N2—C7—H7	121.2		

### Hydrogen-bond geometry (Å, º)

**Table:**

*D*—H···*A*	*D*—H	H···*A*	*D*···*A*	*D*—H···*A*
C7—H7···O1^i^	0.93	2.55	3.296 (4)	137

Symmetry code: (i) *x*+1/2, *y*, −*z*+1/2.
